# Supersolidus liquid phase sintering of water-atomized low-alloy steel in binder jetting additive manufacturing

**DOI:** 10.1016/j.heliyon.2023.e13882

**Published:** 2023-02-20

**Authors:** Mingzhang Yang, Mohsen K. Keshavarz, Mihaela Vlasea, Amin Molavi-Kakhki, Martin Laher

**Affiliations:** a Department of Mechanical and Mechatronics Engineering, University of Waterloo, Waterloo, Ontario N2L 3G1, Canada; b Iron & Titanium, Rio Tinto, Sorel-Tracy, Quebec J3R 1M6, Canada; c Miba Sinter Austria GmbH, Vorchdorf 4655, Austria

**Keywords:** Additive manufacturing, Sintering, Densification, Water-atomization, Low-alloy steel, Binder jetting

## Abstract

This work investigates the feasibility of net shape manufacturing of parts using water-atomized (WA) low-alloy steel with comparable densities to conventional powder metallurgy parts via binder jetting additive manufacturing (BJAM) and supersolidus liquid phase sintering (SLPS). In this study, a modified water-atomized powder grade with similar composition as MPIF FL-4405 was printed and pressure-less sintered under a 95% N_2_–5% H_2_ atmosphere. Combinations of two different sintering schedules (direct-sintering and step-sintering) and three different heating rates (1, 3, and 5 °C/min) were applied to study the densification, shrinkage, and microstructural evolution of BJAM parts. This study demonstrated that, although the green density of the BJAM samples was ∼42% of the theoretical density, the sintered parts experienced large linear shrinkage up to ∼25% and reached ∼97% density without compromising shape fidelity. This was ascribed to a more homogeneous pore distribution throughout the part before ramping up to the SLPS region. The synergistic effects of carbon residue, the slow heating rate, and the additional isothermal holding stage at the solid-phase sintering region were determined to be the key factors for sintering BJAM WA low-alloy steel powders with resulting minimal entrapped porosity and good shape fidelity.

## Graphical abstract

**Figure:**
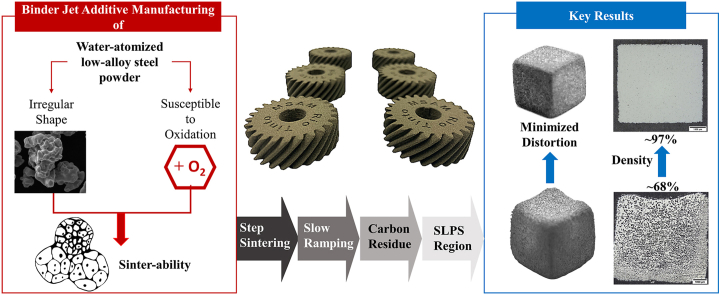

## 1 Introduction

Binder jetting is a powder-bed additive manufacturing (AM) technology that has a remarkable ability to fabricate net shape complex components with relatively lower cost and higher production yield among AM processes [1]. The binder jet AM (BJAM) process relies on selective jetting of fluid binder onto successive layers of powder at near-room temperature to produce designed geometries. The as-printed parts, also referred to as green parts, may undergo debinding and sintering to achieve the desired dimensional, density, and mechanical qualities. To date, numerous metallic alloys have been adopted by BJAM, such as stainless steels, nickel-based alloys, and tool steels to manufacture parts for applications in the automotive, transportation, and aerospace sectors [2]. The gas atomization (GA) process is often used to produce powders for BJAM due to its capacity to generate near-spherical powder particle morphologies and control oxygen content; however, this is a rather costly process when it comes to mass production [3].

To ensure a successful transition to scale-up production and end-use applications, it is essential to develop a low-cost high-performance material system for BJAM. Low-alloy steels are a viable option due to their good strength, ductility, hardness, and wear resistance [4], while also being cost-effective when produced via conventional powder metallurgy (PM) processes. WA powders, which are much less expensive than GA powders, are generally used as feedstock for PM [1,5], but are more irregular in shape with remnant oxides [[6], [7], [8]]. For BJAM, the irregular WA powders require more refined process parameter optimization to address reduced flowability and challenges in powder spreading and layer compaction of the powder bed [9,10]. Moreover, remnant oxides in the form of a surface oxide layer and/or stable oxides can hamper the development of sintering necks and may retain as inclusions that deteriorate the properties of sintered steels [11,12]. Effective strategies for oxide removal learned from the PM industry include the addition of reductive agents (e.g., H_2_ or CO atmosphere) and/or carbon source (e.g., graphite) to reduce surface oxides and prevent their transformation into more thermodynamically stable ones during sintering [13]. The addition of graphite to a BJAM process could be considered an option; however, the additional processing steps to achieve this (likely through mixing) would lead to more complex powder preparation steps, increased costs, and a higher chance of segregation during handling. Interestingly, studies have shown that the deposited binder tends to be retained from de-binding process, resulting in local carbon enrichment (carbon residue) at the particle interfaces [[14], [15], [16]]. The extent to which the carbon residue from the BJAM process influences oxide reduction during sintering is not yet known.

Additionally, unlike traditional PM parts which are pressed to almost 80% relative density before sintering [17,18], BJAM green parts typically have a much lower green part density, typically ranging between 45 and 65% [19], and thus densification from this state usually results in remnant porosity and a significant distortion of design geometry. Prior research has shown that green densities in the range ∼50% are easily obtainable with WA steel powders [3,9]. No literature is currently available that outlines the maximum achievable sintered density in low-green density (≤50%) by BJAM of WA low-alloy steel parts while minimizing geometric distortion.

Several emerging studies have demonstrated the potential to further improve the sintered density of BJAM parts (Inconel [20,21], tool steels [14,15,22], or shape memory alloy [23]) via supersolidus liquid phase sintering (SLPS). Densification and distortion during SLPS depend on the resistance obtained by the structure to viscous deformation, which is related to various metallurgical factors such as grain size, neck size, packing density, and liquid volume fraction [24]. In terms of BJAM WA steel powders, their SLPS behavior has not been explored and can be relatively complex, as the presence of an oxide film may significantly influence the wetting and spreading characteristics needed for densification during SLPS. While there have been reports of achieving 97.5% density with WA stainless steel powders and injection molding process [25], these results cannot be applied to low-alloy steel as stainless steel has higher diffusivity and densification rates due to its dual-phase microstructure at high temperatures [26]. Considering the fundamental sintering mechanisms and based on the understanding of oxide reduction process, this present work looks to conceptualize a two-stage sintering schedule in which the temperature is held for two isotherms. It is hypothesized that such schedule would lead to improved densification for BJAM WA steel.

The objective of this research is to demonstrate the ability to use WA low-alloy steel powder in a BJAM process, along with pressure-less sintering in the SLPS region to manufacture parts with final densities comparable with conventional PM techniques, while retaining geometric shape fidelity in simple cuboid geometries. In this work, newly developed water-atomized low-alloy steel powders were printed via BJAM and sintered under conditions with various isothermal stages and different heating rates to determine the densification behavior (i.e., shrinkage, relative density, pore morphology, and shape loss). The effects of binder residue and remnant oxides were correlated with the resultant densification behavior using microstructural characterizations, differential scanning calorimetry (DSC), thermogravimetric analysis (TGA), and LECO analysis.

## 2 Materials and methods

WA low-alloy steel powders (Rio Tinto Metal Powder, Sorel-Tracy, QC) were used in this study. The chemical composition which was determined by inductively coupled plasma optical emission spectroscopy was given in Table 1. The volume percentile powder size distribution (PSD) and sphericity were analyzed using a particle size and shape analyzer (Camsizer X2, RETSCH). The surface morphology was observed using scanning electron microscopy (SEM) (Vega3, Tescan).

**Table 1:** Chemical composition of the powders studied.

Items	Mn	Mo	C	O	S	P
Content/wt.%	0.20	0.80	0.60	0.074	0.01	≤0.01

The BJAM process was carried out in an BJAM system (M-Flex, ExOne) printer with the following processing parameters: 85 μm layer thickness, recoating speed of 300 mm/s, roller speed of 350 rpm, binder saturation of 35%, drying speed of 15 mm/s, and emitter power of 60%. Green parts were printed into a cuboidal shape of 10 × 10 × 10 mm^3^. For each printing batch, 16 replicates were printed. After printing, the samples were cured at 180 °C for 12 h in a curing oven (CT-333, JPW) under flowing argon, followed by the depowering step.

**Fig. 1:**
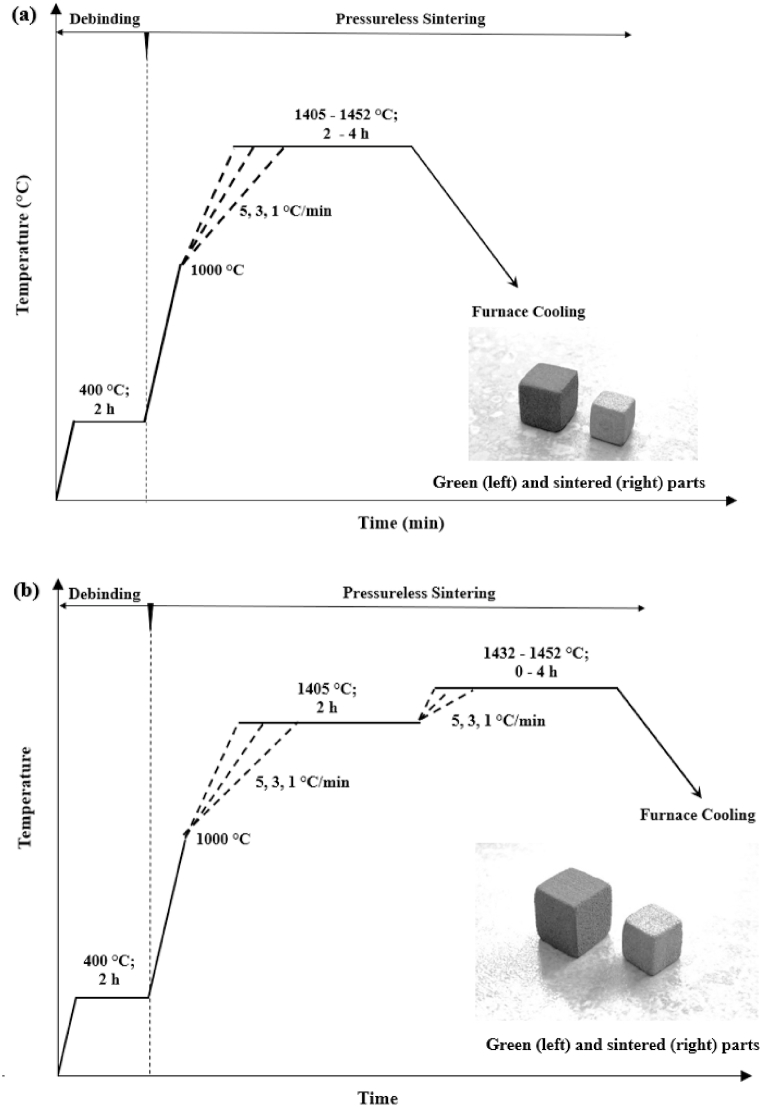
Schematic illustrations of the two applied sintering schedules, with the debinding and sintering under 5% H_2_–95% N2: (a) direct-sintering schedule, and (b) step-sintering schedule.

Thereafter, the specimens were subjected to the debinding and sintering treatments in a tube furnace (GSL-1600X, MTI Corp.) under a 5% H_2_–95% N_2_ atmosphere. The specimens were oriented during sintering in the furnace similarly to the as-printed orientation, with the x-y printing surface coinciding with the horizontal plane and with the z-printing direction aligned with the vertical orientation. Debinding was performed at 400 °C for 2 h (h), while sintering was conducted using two distinct thermal profiles, namely, direct-sintering and step-sintering. Schematic illustrations of the direct-sintering and step-sintering profiles are shown in Fig. 1(a) and (b), respectively. After ramping up to 1000 °C at 10 °C/min, direct-sintering treatment was achieved by gradually heating up to temperatures ranging from 1405 to 1452 °C (i.e., approximate SLPS region) with a ramp rate of 1, 3, or 5 °C/min, hold for up to 4 h, followed by furnace cooling. On the other hand, step-sintering treatment entails three consecutive stages: 1) The samples were firstly heated from 1000 to 1405 °C at 1, 3, or 5 °C/min; 2) after holding for 2 h, the samples were then heated to a temperature between 1432 and 1452 °C at 1, 3, or 5 °C/min; 3) after holding for up to 4 h, the samples were furnace cooled to room temperature.

Characterization of the samples was carried out in both the green and sintered states. The oxygen contents in the green and sintered samples were determined through LECO oxygen analysis (O-836, LECO corporation) conducted at Rio Tinto Metal Powder according to the ASTM E1019. The bulk green density was assessed by measuring the sample volume and mass using a caliper (Digimatic Caliper, Mitutoyo) and precision balance (Secura 225D, Sartorius) with 0.0001 g accuracy, respectively. The sintered density was measured and calculated using the Archimedes method according to ISO 5017 and details were given in Ref. [27]. Phase of selected sintered samples were characterized by X-ray diffraction (XRD) (D8 Diffractometer, Bruker) with Co–K α radiation (λ = 1.79026 Å) operating at 40 kV and 45 mA. The as-sintered samples were polished and etched using 2% Nital for metallographic examination. Imaging of sample cross-sections was conducted with a digital microscope (VHX-700, Keyence). Quantitative measurements of grain size for selected sintered samples were undertaken according to ASTM E112-13. At least 200 grains were measured for each sample. The microstructure and chemical composition of powders in as-received, printed and cured, and sintered states were evaluated using a field-emission SEM (FE-SEM) (Leo 1530, Zeiss) equipped with an energy dispersive X-ray spectroscopy (EDX) detector (Oxford Inc).

DSC and TGA were performed (STA 449 *Jupiter*® instrument, NETZSCH) on the as-received powders and green parts. The experiments were performed under 95% N_2_–5% H_2_ gas flow to simulate the actual sintering atmosphere used in the tube furnace. The temperature program consisted of a heating stage up to 1500 °C with 5 °C/min and a controlled cooling rate of 5 °C/min down to room temperature. The melting point of the as-received powder (with 0.60 wt% C) was estimated using Thermo-calc software with TCFE 10 database.

## 3 Results

### 3.1 Morphology of as-received powders

**Fig. 2:**
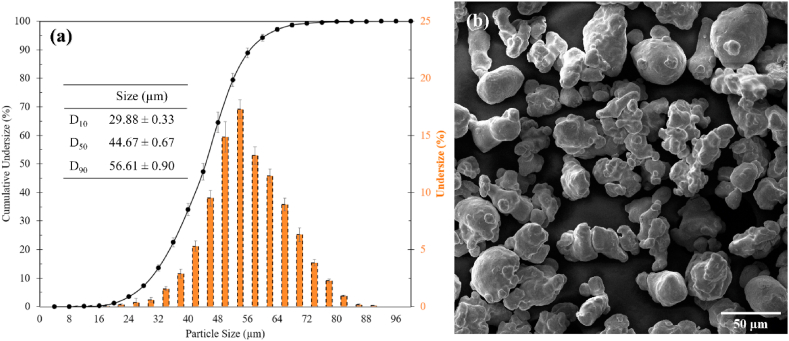
(a) Cumulative volume percentile results for the powder size distribution (PSD) and (b) scanning electron microscopy (SEM) micrograph of the WA powders used in this study.

The PSD result is presented in Fig. 2(a) and shows a D_10_ of 29.88 ± 0.33 μm, a D_50_ of 44.67 ± 0.67 μm, and a D_90_ of 56.61 ± 0.90 μm. This range is within the typical ranges for PM and BJAM applications, respectively. Particle sphericity (lying between 0 and 1, where 1 denotes a perfect sphere) ranges from 0.91 to 0.72 with an average value of 0.75 ± 0.01 μm. Fig. 2 (b) shows a representative SEM micrograph of the as-received powders. Most particles are irregular and angular in shape with small satellites on the surface. These powders have similar morphology as the WA powders previously-studied for BJAM [9]. The D_90_ typically helps to inform the minimum achievable layer thickness in the BJAM process, with the 85 μm selected layer thickness value deemed to be a conservative parameter to ensure print success based on prior works; further layer thickness tuning to improve green part density is possible [19], but considered out of scope for this study.

### 3.2 Densification data

The initial green part density of the cuboid samples was 42.3 ± 0.5%. Table 2 summarizes the relative density, distortion (dimensional shape loss), and percent (%) linear shrinkage of the green parts after sintering from the direct-sintering and step-sintering schedules with 5 °C/min, 3 °C/min, and 1 °C/min heating rates.

**Table 2:** Relative density, shape loss, and linear % shrinkage of the specimens after sintering from different isothermal stages with 5 °C/min, 3 °C/min, and 1 °C/min heating rates.

Heating Rate	Sintering Schedule	Sample Label	Isothermal Stage 1	Isothermal Stage 2	Relative Density	Distortion	x/y Shrinkage	Z Shrinkage
5 °C/min	Direct Sintering	*D1*	1405 °C–2 h	–	67.6%	No	15.3%	18.0%
		*D2*	1432 °C–2 h	–	68.1%	Yes	15.6%	17.6%
	Step Sintering	*S1*	1405 °C–2h	1452 °C–2 h	90.3%	No	23.1%	24.2%
3 °C/min	Direct Sintering	*D3*	1405 °C–2 h	–	66.5%	No	15.3%	17.7%
		*D4*	1452 °C–2 h	–	83.4%	Yes	19.7%	22.1%
	Step Sintering	*S2*	1405 °C–2 h	1432 °C–2 h	74.9%	No	17.7%	20.2%
		*S3*	1405 °C–2 h	1452 °C–2 h	91.9%	No	21.9%	23.0%
1 °C/min	Direct Sintering	*D5*	1405 °C–2 h	–	85.2%	No	21.1%	22.9%
		*D6*	1452 °C–2 h	–	94.9%	No	22.4%	24.9%
		*D7*	1452 °C–4 h	–	96.8%	No	23.0%	25.1%
	Step Sintering	*S4*	1405 °C–2 h	1432 °C–0 h	86.8%	No	21.3%	22.2%
		*S5*	1405 °C–2 h	1432 °C–2 h	87.0%	No	21.8%	22.4%
		*S6*	1405 °C–2 h	1432 °C–4 h	89.0%	No	22.1%	24.7%
		*S7*	1405 °C–2 h	1452 °C–2 h	95.0%	No	22.7%	25.2%
		*S8*	1405 °C–2 h	1452 °C– 4 h	97.1%	No	22.9%	25.3%

With a 5 °C/min heating rate, direct sintering at 1405 °C for 2 (h) (*D*1) resulted in a relatively low final density of 67.6%, yet a further increase in sintering temperature up to 1432 °C led to shape loss and negligible densification (*D2*). Notably, both shape loss and low sintered density were resolved by applying a step-sintering schedule at the same heating rate. For instance, dwelling at 1405 °C for 2 h followed by sintering at a higher temperature led to significant densification of the body up to 90.3% without causing prominent distortion (*S1*).

The sample (*D4*) which was direct sintered at 1452 °C for 2 h with a 3 °C/min heating rate, achieved 83.4% relative density; however, this was accompanied by some distortion. In contrast, step-sintering that consisted of a dwelling at 1405 °C for 2 h, mitigated the distortion at both 1432 °C (*S2*) and 1452 °C (*S3*) sintering temperatures and subsequently, granted further densification to 74.9% and 91.9%, respectively.

When the heating rate was reduced to 1 °C/min, no shape loss was observed in all the specimens, including the specimens sintered up to 1452 °C, implying that they had enough rigidity to sustain densification stresses and gravity during powder consolidation. Under the same final temperatures, the parts sintered at 1 °C/min (*S8*) exhibited a much higher final density compared to the specimens sintered with 3 °C/min (*S3*) or 5 °C/min (*S1*) heating rates. The sintered density depends only on the highest temperature reached and the corresponding holding time, regardless of the isothermal holding at 1405 °C; a longer holding time resulted in a higher final density. When the heating rate was at 1 °C/min and sintering at 1452 °C for 4 h (with or without step-sintering) the highest density of around 97% was achieved (*S8*). This corresponds to a bulk density value of 7.49 g/cm^3^.

### 3.3 Anisotropic shrinkage

An anisotropic shrinkage behavior during sintering was observed, with much larger shrinkage along the z-axis (building direction) than the x/y-axis (Table 2). Owing to the low green density (42%), the specimens underwent a maximum shrinkage up to 25.3% along the z-axis, and 23.1% along the x-/y-axis. The anisotropic sintering behavior is typical to BJAM and can be attributed to factors such as but not limited to the interplay between the BJAM process (preferential powder alignment and powder packing during powder spreading, liquid-powder impact, and fluid imbibition phenomena), the powder properties (powder morphology, electrostatic forces, PSD resulting in varying powder coordination number and contact angle), the cyclic exposure of the material system (local exposure to the liquid binder, heating lamp), as well as geometry, friction and gravity effects [2].

### 3.4 Phase identification and grain size

**Fig. 3:**
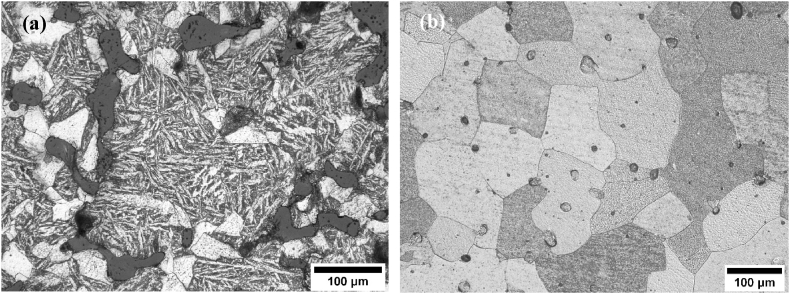
Optical micrographs of (a) sample *D2* and (b) sample *S7*. Porosities are shown as black/dark areas.

Representative microstructures of samples with low sintered density (*D2* – 68.1% relative density) and high sintered density (*S7* – 95.0% relative density) are presented in Fig. 3(a) and (b), respectively. Sample *D2* consists of a heterogeneous distribution of complex phases including polygonal ferrite, Widmanstätten ferrite, and pearlite. In contrast, sample *S7* consists of polygonal ferrite and pearlite with much larger grain sizes. Notably, similar coarse-grained microstructure was observed in all the samples with densities above 85%.

**Fig. 4:**
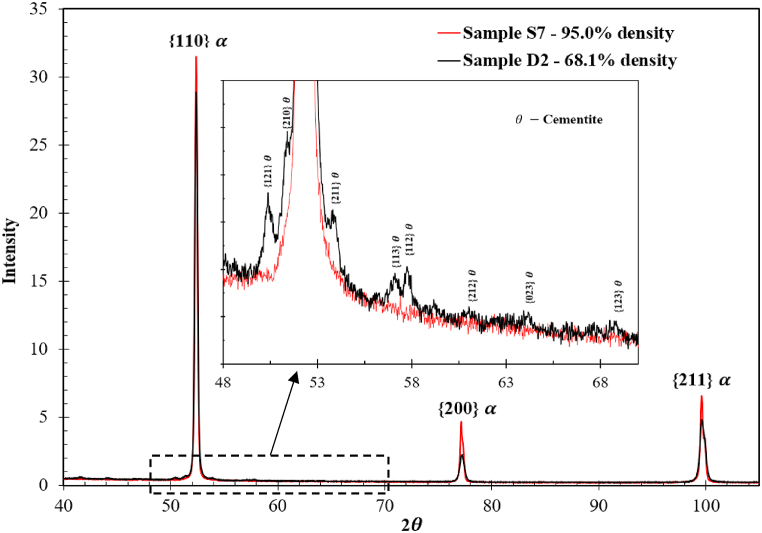
XRD diffraction patterns obtained from sample *D2* and sample *S7*.

The corresponding XRD patterns of sample *D2* and sample *S7* are shown in Fig. 4(a) and (b), respectively. The *α-*Fe phase was identified in both patterns. The trace of cementite was only detected in *D2*, the sample with low sintered density. Specifically, the peaks for θ-cementite are shown in enlarged in the selected area. Associated with the presence of cementite is a broadening of full width at half maximum (FWAM) of {110} *α-*Fe peak, indicating that sample *D2* has a slightly higher degree of elastic strain caused by the lattice misfit between *α-*Fe and cementite.

**Fig. 5:**
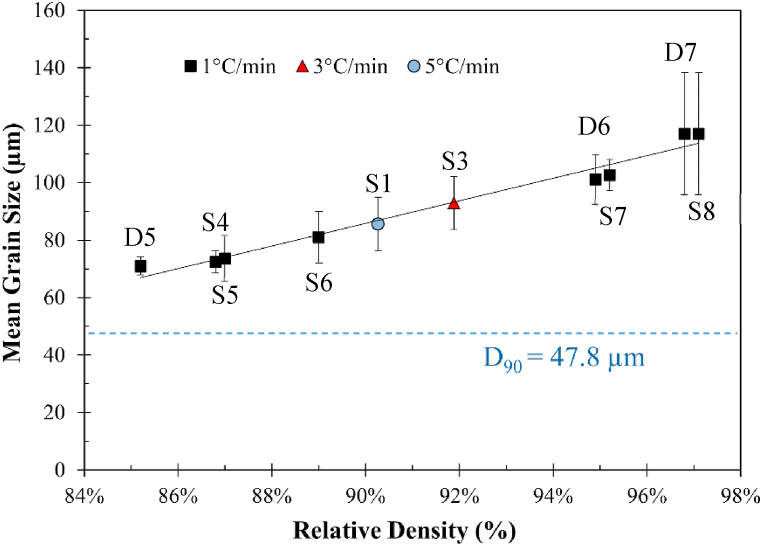
Mean grain size versus relative density.

Fig. 5 shows the mean grain size vs the relative density. The grain size of the parts with densities lower than 85% was not measured due to the complexity of the microstructure. Grain size is linearly proportional to the relative density with an R-squared value of 0.991, indicating that the densification after reaching 85% density was solely governed by grain growth densifying mechanism. Fundamentally, grain growth is an interface energy annihilation process correlated to the inverse square root of porosity percentage [28]. As a result, pore removal is the determining factor for further increasing density beyond 85%.

Remarkably, the smallest mean grain size (70.5 μm among all samples with density above 85%), which belonged to the sample *D5*, was still much larger than the D_90_ (47.8 μm) of the as-received powders, meaning that the necking of particles was completed before reaching 85% density for grain growth to occur. This finding is in accordance with the theory of German [29], where the peak grain boundary area occurs near 85% relative density.

### 3.5 Pore morphology

**Fig. 6:**
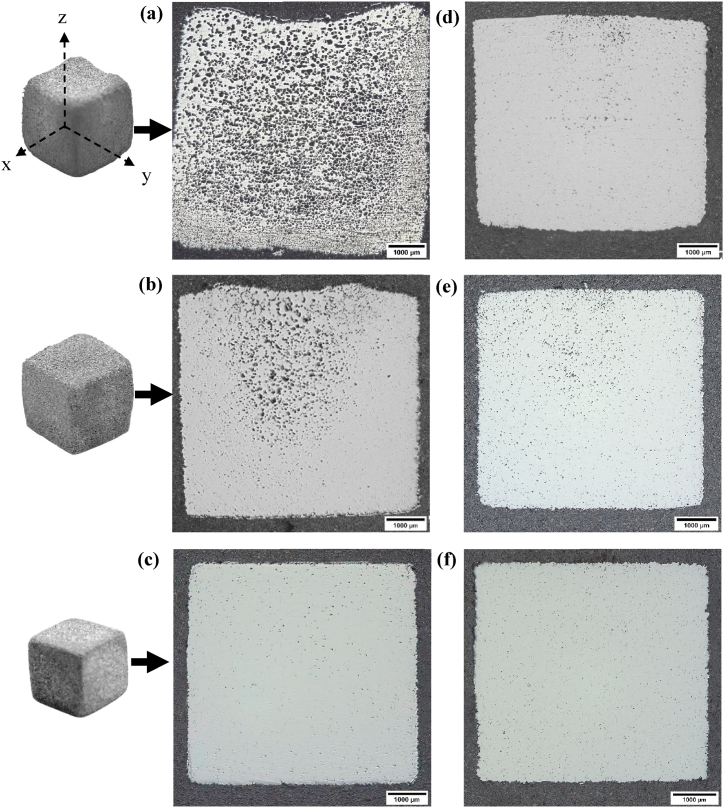
Optical micrographs of samples (a) *D2 –* 68.1% density, (b) *D4 –* 83.4% density, and (c) *D7 –* 96.8% density sintered from the direct-sintering schedule, and samples (d) *S1 –* 90.3% density, (e) *S3 –* 91.9% density, and (f) *S8 –* 97.1% density sintered from the step-sintering schedule.

Fig. 6(a)–(c) show the optical micrographs of the direct-sintered specimens *D2*, *D4*, and *D7*, respectively, cut in the zy-plane. Sample *D2* sintered with a 5 °C/min heating rate experienced severe slumping and a low degree of densification (68.1%), which can be related to the presence of the large and densely distributed pores that are shown in Fig. 6(a). Sample *D4* sintered at a slower heating rate of 3 °C/min resulting in overall better pore closure and densification, as seen in Fig. 6(b). Nevertheless, a large fraction of large pores with non-uniform distribution were found in the structure, which in turn led to prominent geometric distortion. A further decrease in heating rate to 1 °C/min was shown to be effective in eliminating the remnant large pores and additional densification, as seen in Fig. 6(c).

Fig. 6 (d), (e), and (f) show the optical micrographs of the step-sintered specimens *S1*, *S3*, and *S8*, respectively, cut in the zy-plane. The remarkable improvement in sintered density using a step-sintering schedule compared to direct-sintering schedule (Table 2) can be mainly characterized by the significant reduction in large pores. Specifically, step-sintering at 5 °C/min and 3 °C/min removed a vast majority of large pores, resulting in better shape retention and enhanced density to over 90% (see Fig. 6(d) and (e) respectively); the non-uniform distribution of pores was also improved based on visual inspection. It is noteworthy that the sintering behavior (i.e., shape retention, pore size and distribution, and density) of direct-sintered samples at 1 °C/min was quite similar to the step-sintered counterparts; both sintering schedules can produce equally dense and homogeneous structure while maintaining structural rigidity for the given geometries.

### 3.6 Phase transformation upon sintering

The distinct sintering behaviors resulting from varying heating rates and isothermal stages discussed above are quite intriguing, since the onset of distortion was expected to rely only on the sintering temperature. Potentially, the liquid form within the experimental sintering temperature range can be related to these observations. Hence, DSC and TGA were carried on the green part and as-received powder to elucidate the underlying mechanisms.

**Fig. 7:**
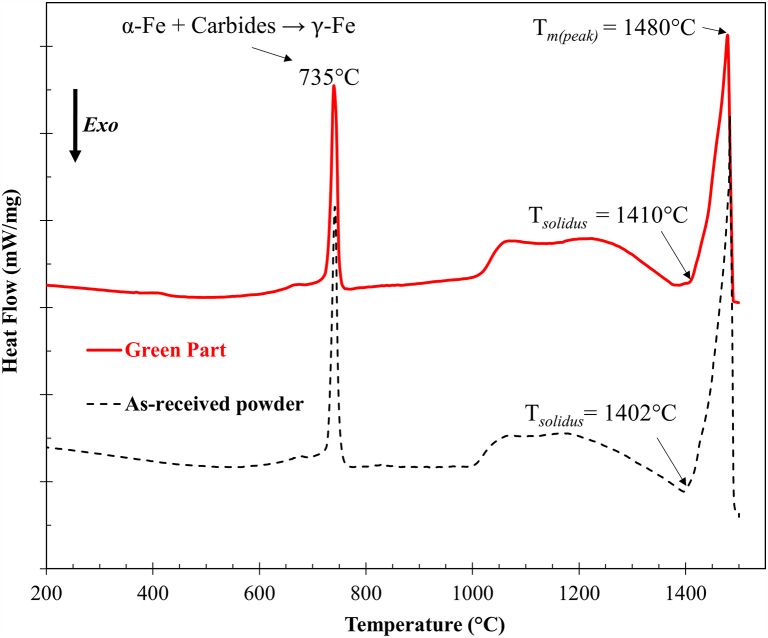
DSC curves of the green part and as-received powders. (For interpretation of the references to colour in this figure legend, the reader is referred to the Web version of this article.)

Fig. 7 shows the DSC curves of the green part and as-received powders. Heating to around 735 °C revealed a sharp endothermic peak that corresponds to the transformation of α-Fe and precipitated carbides to γ-Fe. In addition, the DSC result identified the solidus temperature (*T*_*solidus*_) of green part (∼1410 °C) is slightly higher than that of as-received powder (∼1402 °C). This highlights the possibility of compositional changes during printing, curing, and sintering.

The measured *T*_*solidus*_ values (i.e., 1402 °C for the as-received powder, 1410 °C for the green part) are reasonably well predicted as the *T*_*solidus*_ by the equilibrium calculation via Thermo-calc (i.e. 1408 °C). This indicates that the actual carbon content in both green part and as-received powders before reaching *T*_*solidus*_ was approximately 0.60 wt%. Furthermore, the measured *T*_*m(peak)*_ for both materials (1480 °C) are consistent with the equilibrium calculated liquidus temperature (1482 °C). The equilibrium liquid volume fractions at the isothermal temperatures of 1405 °C, 1432 °C, and 1452 °C, which were used in this study, were determined using Thermo-calc and found to be approximately 0 vol%, 12 vol%, and 33 vol%, respectively.

**Fig. 8:**
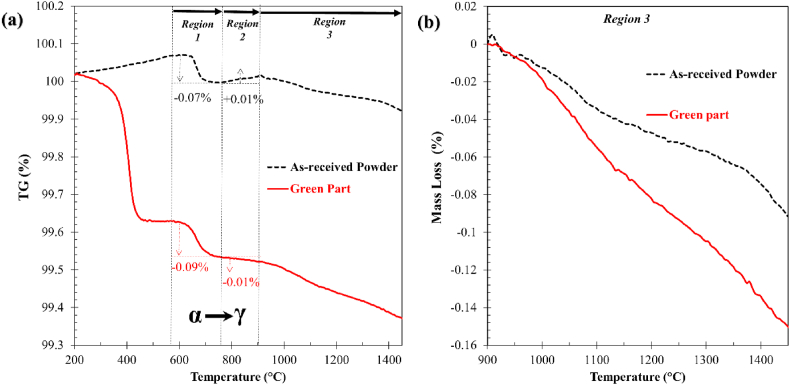
(a) TGA curves of as-received powder and green part; (b) mass loss % of as-received powder and green part in Region 3. (For interpretation of the references to colour in this figure legend, the reader is referred to the Web version of this article.)

Fig. 8 compares the TGA curves of the green part and as-received powder. The rapid weight loss in green parts from 300 to 560 °C corresponds to the decomposition of binders (Fig. 8 (a)). The as-received powders experience 0.04% mass gain, pointing toward a material that is prone to oxidation at low temperatures (<560 °C). Further heating revealed three other regions of weight changes, i.e., Region 1 (560–735 °C), Region 2 (735–900 °C), and Region 3 (>900 °C).

In Region 1, the mass losses for both materials are approximately the same in terms of onset temperature and magnitude (Fig. 8(a)). The mass losses flatten out at the α→γ transformation temperature, indicating a strong phase dependence.

Region 2 revealed a clear deviation in mass change between the two materials (Fig. 8(a)). The as-received powder experienced a mass gain of around 0.01%, as opposed to the green part which experienced a 0.01% mass loss. This implies that within this narrow temperature range, the green part is less sensitive to oxidation and more prone to certain reduction reactions.

This distinct mass loss behavior is further evidenced in Region 3 (Fig. 8(b)), where the weight loss corresponding to the green part (0.154%) is much higher than that of the as-received powder (0.086%). It can be deduced that additional reducing agent(s) may have been introduced into the system during the BJAM process and increased the reduction potential at high temperatures (Regions 2 and 3). In addition, multiple underlying reduction mechanisms may have taken place in parallel.

## 4 Discussion

Previous studies on the surface of iron-based alloy powders produced by WA and annealing indicated the presence of heterogeneous oxide layers, consisting of oxide particulates formed by strong oxide formers such as Cr, Si, and especially Mn (the dominant cation), and iron oxide layer in between [30,31]. LECO analyses of the powders in the as-received and green states showed an increase in oxygen content from 0.074 to 0.170 wt% (Table 3), indicating the oxidation of low-alloy steel powders may occur during the printing and curing. The deposited aqueous binder (composed of ethanol, 2-Butoxyethanol, and water), the applied cyclic heating/drying during printing, and the oxygen impurity in the curing oven can contribute to this oxidation.

**Table 3:** Oxygen content (wt.%) in the powders and BJAM parts at different states.

As-received powder	Green part	Part heated to 1000 °C	Part heated to 1432 °C
0.074	0.170	0.153	0.092

**Fig. 9:**
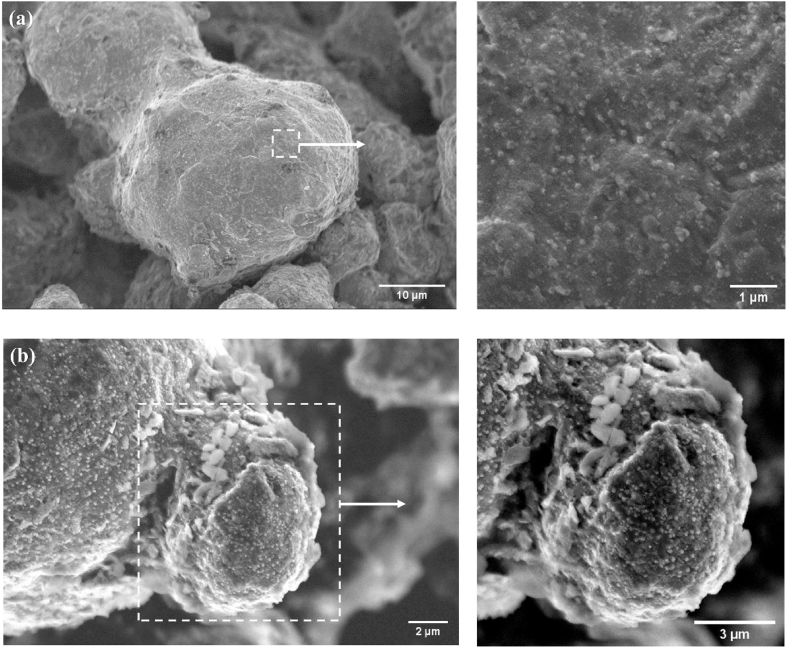
Secondary electron (SE)−SEM micrographs of the surfaces of (a) as-received powders and (b) powders in green state.

Fig. 9(a) and (b) show the surface conditions of the powders in the as-received and green states, respectively. Nanosized oxide particulates (<30 nm) with a spherical shape were visible on the surface of both powders. Oxide particulates were readily scattered on the near-spherical powder particle with smaller diameters owing to their higher surface-to-volume ratio (i.e., higher tendency to oxidation) [31]. In addition, a few large particulates (>500 nm) were observed in the inter-particle neck regions but more rarely, as seen in Fig. 9(b). It has been reported that these large oxide particulates arise from the agglomeration of finer ones [32]. Unfortunately, due to the limited resolution of SEM-EDX, no visible oxide layer around the as-received powders was detected in this study. Nevertheless, the study by Hryha and Gierl et al. can serve as a reasonable estimate of the thickness of the oxide layer - for WA powders with 0.3–1.8 wt% Mn and 0.08–0.16 wt% O, more than 90% of the powder surface was covered by iron oxide layers with a thickness of 6–7 nm [30].

**Fig. 10:**
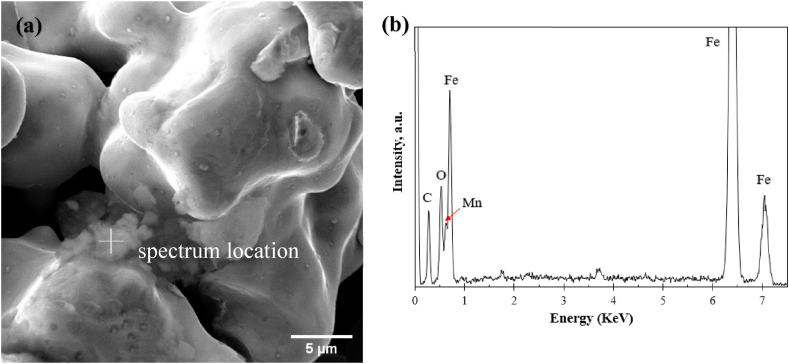
(a) SE−SEM micrographs of the inter-particle connections of the part pre-sintered at 1000 °C-0 h; (b) EDX spectrum of the particulate features at the particle interface.

From Table 3 the oxygen content reduced partially from 0.170 to 0.153 wt% after heating to 1000 °C. This reduction is illustrated in the fractography (Fig. 10(a)) where the powder particle surface became cleaner and contained fewer numbers of oxide particulates compared to the powders in green state (Fig. 9(b)). In addition, the signs of inter-particle neck formation were identified. The overall reduction of oxides in this stage was reflected by the weight loss in Region 1 (Fig. 8), in which the following two reduction mechanisms may occur: oxide dissociation (Equation (1)) and reduction by H_2_ (Eq. (2)) [33]:

(1) $$\frac{2}{y}M_{x}O_{y}⇌\frac{2x}{y}M+O_{2}$$

(2) $$\frac{2}{y}M_{x}O_{y}+2H_{2}⇌\frac{2x}{y}M+2H_{2}O$$

where M and M_x_O_y_ denote the metal and its oxide, respectively. The mass loss due to the above two reactions is expected to be related to the removal of both surface and internal oxides. This was observed by the decrease in mass loss rate after α→γ transformation (Region 2 in Fig. 8), in which *α*-Fe with BCC crystal structure allows for rapid interstitial solid-state diffusion that facilitates mass transport and subsequent oxide removal [34].

In addition, the presence of oxide agglomerates was occasionally found in the necking area between two neighboring particles; an example is presented in Fig. 10(a) and (b). The EDX spectrum in Fig. 10(b) shows that, in addition to oxygen content, the agglomerates are rich in carbon compared to the surrounding area. This is believed to be the carbon residue generated from binder decomposition that remained un-dissolved even after heating up to 1000 °C. In light of the work of Chasoglou and Hryha et al. [33], iron oxides and undissolved graphite still appeared after heating up to 1120 °C under 10% H_2_–N_2_. Associated with this local carbon enrichment are two other reduction mechanisms: direct carbothermal reaction (Eq. (3)) and indirect carbothermal reaction (Eq. (4)) [33]:

(3) $$\frac{2}{y}M_{x}O_{y}+2C⇌\frac{2x}{y}M+2CO$$

(4) $$\frac{2}{y}M_{x}O_{y}+2CO⇌\frac{2x}{y}M+2CO_{2}$$

It is noteworthy that the above two reactions are thermodynamically favorable above Boudouard equilibrium temperature (∼720–800 °C) as the reducing activity of undissolved carbon increases [35], but they become more active above 900 °C [36,37]. The above carbothermal reactions can be reflected by the higher mass loss rate of green part compared to the as-received powder (Region 3 in Fig. 8) as well as the substantial reduction in oxygen content from 0.153 to 0.092 wt% after heating from 1000 °C to 1432 °C (LECO analysis presented in Table 3).

Fig. 10 (a) also shows that many oxide particulates were locked inside the sinter necks (the brighter contrast). Upon sintering at higher temperatures and longer sintering time, more intensive development of necking is expected to cause more oxide particulates enclosed within the semi-opened or close (isolated) porosity. This would further complicate the necking and subsequent densification behavior of the powder compact. Specifically, the reducing conditions provided for the open pores near the surface of the sample and at the interior of semi-opened or isolated pores are completely different due to the different rates of gas replenishment and removal of reduction products [35]. It is known that the occurrence of the above oxide reduction reactions (Eqs. (2), (3), (4)) decisively depends on the partial pressure of H_2_/H_2_O (effective dew-point) and CO/CO_2_ rather than the actual H_2_ content in the atmosphere [33]. Therefore, the accumulation of the reaction products (e.g., CO_2_, H_2_O) in the semi-open or close pores retards further reduction process.

In contrast, the efficient outflow of reaction products increases the reducing potential of the sintering atmosphere. Furthermore, the continuous replenishment of H_2_ can further increase the reducing potential by the regeneration of CO through ‘‘water-gas’’ reaction (typically occurs at high temperatures (>900 °C)) [38], as described by Eq. (5):

(5) $$H_{2}+CO_{2}⇌H_{2}O+CO$$

In light of the difference in reducing potential at different sites of part, the applied heating rate can directly affect the extent of oxide removal and may influence densification behavior and gas entrapment. It can be deduced that ramping at a higher heating rate to above *T*_*solidus*_ could lead to the entrapment of remnant oxides and reduction vapor products inside pores due to the rapid closure of the surrounding pore channels upon liquid formation.

**Fig. 11:**
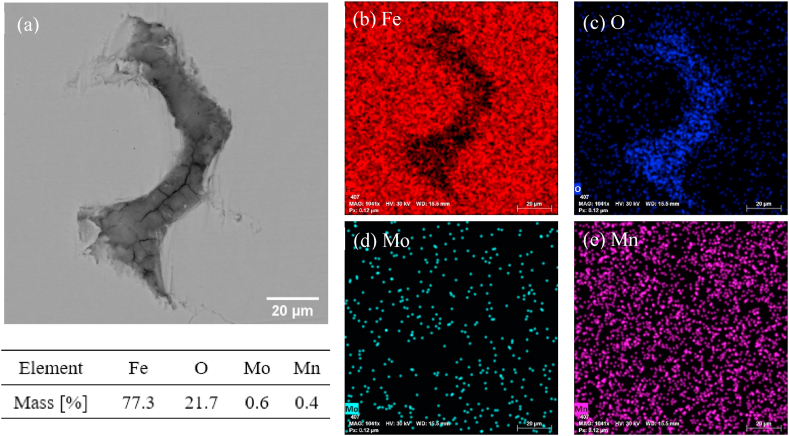
(a) Backscattered Electron (BSE) – SEM micrographs of the direct-sintered sample (*D*2) from 1432 °C to 2 h with 5 °C/min, and the corresponding elemental maps of (b) Fe, (c) O, (d) Mo, and (e) Mn collected by EDX.

Evidence of the retained oxides due to sintering at SLPS region with a high heating rate (i.e., 5 °C/min) was evidenced in SEM-EDX analysis. An example is shown in Fig. 11(a–e), where oxides were found at the interparticle neck region in sample *D2*; this of course retarded densification by acting as a diffusion barrier. Notably, some cracks were also found on the oxide layer, which can be attributed to the thinning of oxide film due to the presence of liquid phase that results in dissolution of oxygen in the liquid to re-combine with high oxygen affinity elements (e.g., Mn) [39,40]. Meanwhile, as a result of the high heating rate, reaction vapor products such as CO, H_2_O, and CO_2_ that are nearly insoluble in iron-FCC can remain entrapped within pores and lead to pore enlargement through Ostwald ripening as a result of grain growth [41] or particle rearrangement [24]. Based on the finding of Ghasemi and Azadbeh et al. [42], large pores migrated to the top of samples due to buoyancy force, while the liquid settled to the bottom area due to gravity. The resultant heterogeneous distribution of pore and liquid phase can cause geometric distortion of the part upon densification. In addition, the large and stabilized pores can lead to swelling. All the above phenomena (i.e., pore enlargement, heterogeneous pore distribution, and distortion) due to high heating rates were evidenced in Fig. 6(a)–(c).

**Fig. 12:**
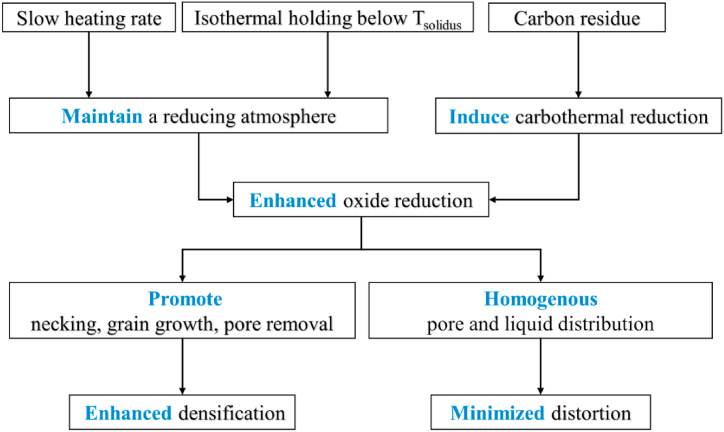
Effects of carbon residue, heating rate, and step-sintering on the densification behavior of BJAM WA steel.

A mechanism is proposed and schematically illustrated in Fig. 12 to describe the synergistic effects of carbon residue, heating rate, and step-sintering on the densification of BJAM WA steel. From Table 2 the highest attainable density by sintering at temperatures below *T*_*solidus*_ (1408 °C) was 85.2%. As a rule of thumb, open pores close as porosity declines, typically starting at 85% density and reaching full closure by 95% density [43]. Therefore, a slow heating rate (1 or 3 °C/min vs 5 °C/min) or step-sintering with an isothermal holding at a temperature (i.e., 1405 °C) lower than *T*_*solidus*_ allowed more residual oxides to be reduced under a ‘‘replenishing’’ reducing atmosphere before the closure of the continuous network of open porosity. Similarly, the carbon residue from binder decomposition helped to reduce oxides at the high-temperature range (>960 °C) by participating in carbothermal reduction (Eqs. (3) and (4)). The enhanced oxide reduction facilitated necking and the subsequent grain growth and pore removal, which were deemed to be the determining factors for further densification above 85% (Fig. 5). Meanwhile, the removal of large and stabilized pores resulted in a macrostructure with a more homogeneous pore distribution, which in turn enabled densification up to 97.1% while retaining geometric shape fidelity during SLPS (Table 2).

It is important to note that the reliable and successful applications of low-alloy steels in structural applications are possible only if sintered relative densities of 92% can be ensured [44]. In this perspective, the results of present approach (i.e., SLPS in combination with a two-stage sintering schedule) are significant, as they successfully address the concern that BJAM and WA low-alloy steel powders are not amenable to producing high-density parts in this work.

## 5 Conclusion

This study examines the ability to sinter WA low-alloy steel fabricated by BJAM process to a relative density of 97.1% via supersolidus liquid phase sintering (SLPS) with minimal shape loss. A comprehensive investigation was completed to identify any potential challenges in the densification of oxygen-containing steel powders with a significantly lower green density compared to traditional PM green parts, which are typically around 80%. The following major conclusions are drawn from the present study.

1] The highest attainable density by sintering at temperatures below *T*_*solidus*_ was 85.2%. As relative density increased further above 85% using SLPS, pore removal and the subsequent grain growth became the dominant densifying mechanism.
2] The best sintering conditions for achieving higher density and minimized distortion involve a slower heating rate (≤3 °C/min) and/or an isothermal holding below *T*_*solidus*_ before ramping up to SLPS region. In these conditions, more oxides can be reduced by carbon residue from binder decomposition and H_2_ from the sintering atmosphere, leading to a macrostructure consisting of homogeneously distributed pores with smaller sizes before liquid formation. The improved pore morphology enables the parts to be further densified at higher temperatures.
3] Owing to the low green density (42%), the densification up to 97.1% corresponded to the linear shrinkage of ∼25% on the z-axis, and ∼23% on the x/y-axis. Nevertheless, the surprisingly large shrinkages have a negligible effect on the geometrical precision of parts for the geometries used in this study due to an improved pore morphology using optimized sintering conditions.

In the future, a systematic investigation of the processing window of BJAM WA low-alloy steel parts with larger dimensions and more complex geometry will be undertaken.

## Author contribution statement

Mingzhang Yang: Conceived and designed the experiments; Performed the experiments; Analyzed and interpreted the data; Wrote the paper.

Mohsen K. Keshavarz: Conceived and designed the experiments; Wrote the paper.

Mihaela Vlasea: Conceived and designed the experiments.

Amin Molavi-Kakhki and Martin Laher: Conceived and designed the experiments; Contributed reagents, materials, analysis tools or data.

## Funding statement

Assistant Prof. Mihaela Vlasea was supported by Federal 10.13039/501100006266Economic Development Agency for Southern Ontario, Canada [814654].

## Data availability statement

Data will be made available on request.

## Declaration of competing interest

The authors declare that they have no known competing financial interests or personal relationships that could have appeared to influence the work reported in this paper.
